# TIMP1 secretion induced by *Toxoplasma* effector GRA24 via p38 MAPK signaling promotes non-disruptive parasite translocation across polarized brain endothelial monolayers

**DOI:** 10.1128/msphere.00102-25

**Published:** 2025-04-23

**Authors:** Elena Afanaseva, Antonio Barragan

**Affiliations:** 1 Department of Molecular Biosciences, The Wenner-Gren Institute, Stockholm University 193207, Stockholm, Stockholm County, Sweden; Virginia-Maryland College of Veterinary Medicine, Blacksburg, Virginia, USA

**Keywords:** (MeSH): blood–brain barrier, intestine, biological barrier, Apicomplexa, Coccidia, host-pathogen interactions

## Abstract

The protozoan *Toxoplasma gondii* first crosses the intestinal wall and then the blood–brain barrier (BBB) to establish chronic, latent infections in humans and other warm-blooded vertebrates. However, the molecular mechanisms underlying this stealthy colonization remain poorly understood. In this study, we investigated the passage of *T. gondii* tachyzoites across polarized monolayers of murine brain endothelial cells (bEnd.3) and human intestinal cells (Caco-2). We found that exposure to live *T. gondii* tachyzoites, but not to tachyzoite lysate or lipopolysaccharide, induced elevated transcription and secretion of tissue inhibitor of metalloproteinases 1 (TIMP1), a pleiotropic protein linked to BBB maintenance. Recombinant TIMP1 consistently increased *T. gondii* transmigration across monolayers, while pharmacological inhibition of matrix metalloproteinases (MMPs) non-significantly impacted transmigration. Through a combined approach of pharmacological inhibition and mutant *T. gondii* lines, we identified the MYR translocon-associated effector GRA24 and host cell p38 mitogen-activated protein kinase (MAPK) signaling as key mediators of *Timp1* induction. Moreover, despite *T. gondii* transmigration, cell polarization and barrier integrity were preserved, suggesting a non-disruptive passage of tachyzoites with minimal or transient barrier dysregulation. These findings reveal a role for GRA24-p38 MAPK signalling and TIMP1’s MMP-independent effects in facilitating the translocation of *T. gondii* across restrictive biological barriers.

**IMPORTANCE:**

The parasite *Toxoplasma gondii*, which is globally widespread, colonizes the brains of humans and other warm-blooded animals. To do so, it first crosses the gut wall before entering the brain via the bloodstream. However, the mechanisms by which *Toxoplasma* overcomes the body’s restrictive biological barriers remain largely unknown. In this study, we used cellular models of the gut and brain barriers to investigate how the parasite passes through. We found that *Toxoplasma* induces cells to secrete TIMP1, a multifunctional protein that reduces inflammation and is linked to blood–brain barrier protection. Surprisingly, TIMP1 also facilitated *Toxoplasma*’s passage across cellular barriers. This elevated TIMP1 production and secretion by host cells was triggered by a secreted *Toxoplasma* effector protein (GRA24) and mediated through host cell signaling pathways (p38 MAPK). These findings suggest that *Toxoplasma* manipulates host cells to produce factors that aid its colonization while suppressing inflammation.

## INTRODUCTION

In vertebrates, biological barriers protect organs from external insults and help maintain tissue homeostasis. The intestinal barrier and the blood–brain barrier (BBB) are particularly crucial, as they not only physically separate diverse body compartments but also share physiological similarities (1). Both gut epithelial cells and brain endothelial cells are connected by tight junctions (TJs) that restrict and regulate passage. Microorganisms have evolved strategies to thrive at these physiological interfaces, and several pathogens possess the ability to circumvent the restrictive nature of biological barriers for host colonization (2, 3).

The apicomplexan parasite *Toxoplasma gondii* is chronically carried by a significant portion of warm-blooded vertebrates, including humans (4). Three clonal lineages of *T. gondii* (types I, II, and III) predominate in humans and animals in Europe and North America (5). Following oral infection through contaminated food or water, *T. gondii* tachyzoite stages replicate within the intestinal wall before undergoing rapid systemic dissemination via the circulatory system (6). Ultimately, *T. gondii* reaches the central nervous system (CNS), where it forms latent cysts. Although chronic carriage of *T. gondii* is generally considered asymptomatic, acute primary infection or reactivation can lead to severe neurological disease in developing fetuses and life-threatening encephalitis in immunocompromised individuals, respectively (7).

*T. gondii* tachyzoites utilize gliding motility to actively invade virtually any nucleated cell (8) and to migrate within tissue microenvironments (9). Consequently, tachyzoites can transmigrate across polarized epithelial and endothelial cell monolayers *in vitro* (10, 11). In the parasite’s journey to form brain cysts, rapid traversal of intestinal cellular barriers is likely advantageous for initial systemic dissemination, while passage across the BBB is crucial for colonizing the CNS (12). Additionally, focal infections of the intestinal epithelium and cerebral endothelial cells occur during *T. gondii* organ colonization (9, 13–15). From its intracellular replicative niche, *T. gondii* exports effector proteins into the host cell nucleus and cytosol primarily via the MYR secretory machinery (16), enabling the modulation of host cell gene expression and signaling (17).

Inflammatory conditions, such as infections, can jeopardize the homeostatic and restrictive functions of the intestine and the BBB (18, 19). Immune activity by resident cells and infiltrating leukocytes is essential for preventing colonization or eradicating invading pathogens (20); however, this immune response can also compromise barrier function (19, 21). Therefore, it is crucial to strike a balance between controlling infection and limiting excessive inflammation (22, 23). The tissue inhibitors of matrix metalloproteinases (TIMPs) consist of four members (TIMP1-4), which tightly regulate the activity of matrix metalloproteinases (MMPs) during inflammation (24, 25). Recent findings, however, have attributed pleiotropic, MMP-independent roles to TIMPs—particularly TIMP1—acting as modulators of various cellular functions, including angiogenesis, differentiation, oncogenesis, and signaling with cytokine-like effects (26, 27). The expression of TIMPs is transcriptionally and post-transcriptionally regulated by an array of growth factors, cytokines and chemokines (28).

Thus, the intestinal epithelium and cerebral endothelium can serve as focal points of infection by *T. gondii*. However, there is limited understanding of the factors that mediate the colonization of deeper tissues by the parasite. Furthermore, elevated levels of TIMP1 have been detected in the serum and brain tissue of *T. gondii*-infected mice (15, 29), with TIMP1-deficient mice exhibiting reduced cerebral parasite loads (30). Consequently, we investigated the role of TIMP1 in the passage of *T. gondii* across polarized epithelial and endothelial monolayers.

## RESULTS

### TIMP1 expression and secretion by brain endothelial cells and intestinal epithelial cells upon challenge with *T. gondii*

**Fig 1:**
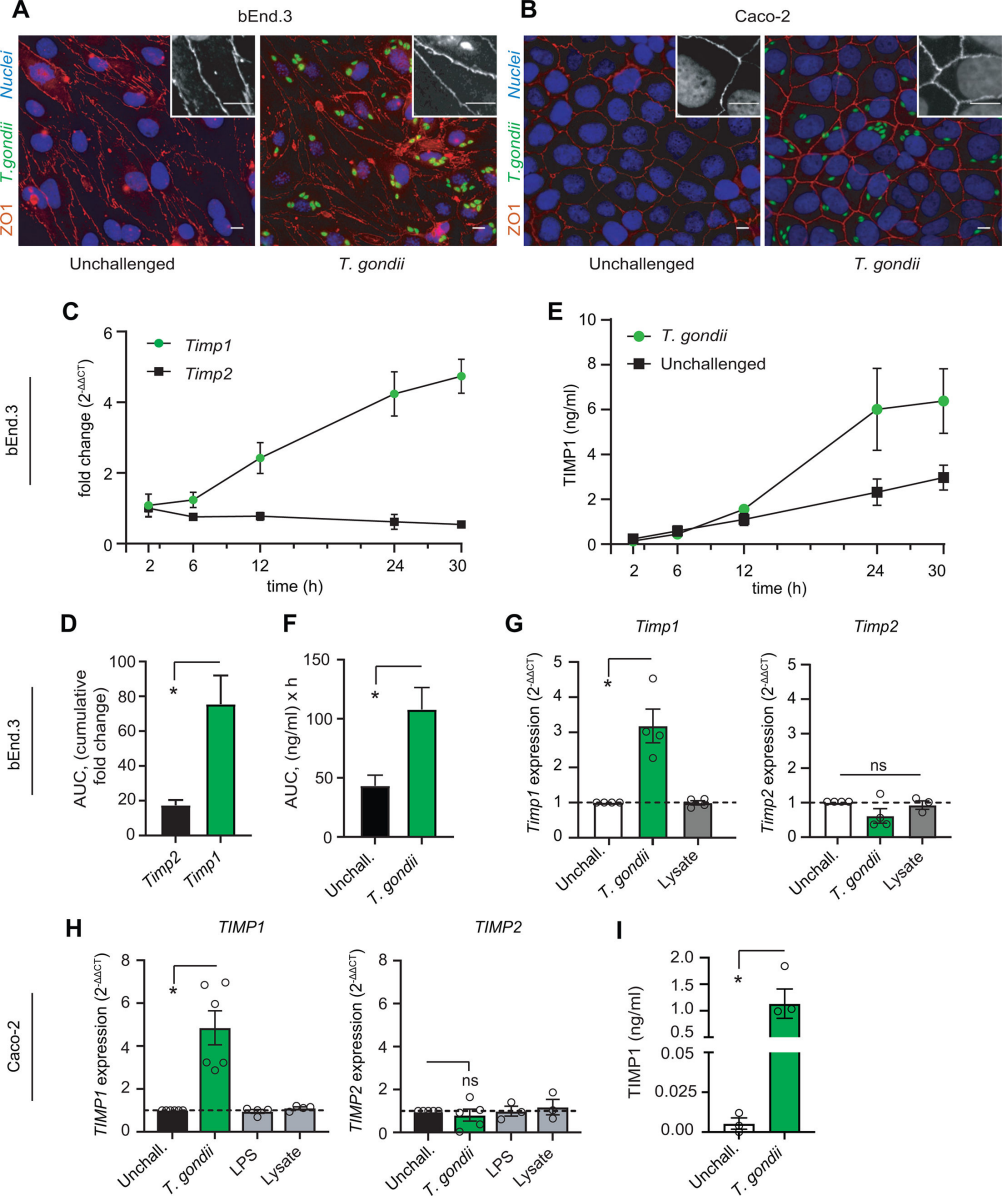
TIMP1 expression and secretion by epithelial and endothelial cells upon challenge with *T. gondii.* (**A and B**) Representative micrographs of confluent bEnd.3 (**A**) and Caco-2 (**B**) monolayers challenged with GFP-expressing *T. gondii* tachyzoites (RH-LDM, green) and stained for the TJ protein ZO-1 (red), with DAPI-stained nuclei (blue), scale bar = 20 µm. Insets show magnification of ZO-1 staining, scale bar = 10 µm. (**C**) qPCR analyses of *Timp1* and *Timp2* cDNA from bEnd.3 cells challenged with *T. gondii* tachyzoites (RH-LDM) for 2 h (MOI 8), 6 h (MOI 4), 12 h (MOI 2), 24 h (MOI 2) and 30 h (MOI 1). (**D**) Area under the curve analysis of the graphs displayed under (**C**). Bars indicate the cumulative fold change of *Timp1* and *Timp2* genes ± SE for the first 24 h. (**E**) Abundance of TIMP1 polypeptide (ng/mL) in supernatants from bEnd.3 cells challenged with *T. gondii* tachyzoites (RH-LDM) for 2 h (MOI 8), 6 h (MOI 4), 12 h (MOI 2), 24 h (MOI 2), and 30 h (MOI 1), determined by ELISA. (**F**) Area under the curve analysis of the graphs displayed under (**E**). Bars indicate the cumulative secretion of TIMP1 protein ± SE. (**G**) qPCR analyses of *Timp1* and *Timp2* cDNA from bEnd.3 cells challenged with *T. gondii* tachyzoites (RH-LDM, MOI 2) or parasite lysate (equal to MOI 2) for 24 h. (**H**) qPCR analyses of *TIMP1* and *TIMP2* cDNA from Caco-2 cells challenged with *T. gondii* tachyzoites (RH-LDM, MOI 2), LPS (10 µg/mL) or parasite lysate (MOI-equivalent 2) for 2 h. (**I**) Abundance of TIMP1 polypeptide in supernatants of Caco-2 cells challenged with *T. gondii* tachyzoites (RH-LDM) for 24 h (MOI 2) or unchallenged (Unchall.), determined by ELISA. Data are presented as mean (±SEM) from three to six independent experiments per condition. qPCR data are displayed as fold change (2^−ΔΔCt^) in relation to unchallenged condition. Statistical comparisons were performed with permutation test (**D and F**), repeated measures analysis of variance, Sidak post hoc test (**G**), mixed-effect model (**H**), and Student’s *t*-test (**I**), *P < 0.05, ns: non-significant

To investigate the potential impact of *T. gondii* infection on TIMP1 in cellular barriers, confluent monolayers of murine brain endothelial cells (bEnd.3) (Fig. 1A) and human gut epithelial cells (Caco-2) (Fig. 1B) were challenged with freshly egressed *T. gondii* type I (RH) tachyzoites. Interestingly, a sustained elevation of *Timp1*, but not *Timp2*, mRNA expression was detected in bEnd.3 monolayers (Fig. 1C and D). Correspondingly, increased amounts of TIMP1 protein were measured over time in supernatants of parasite-challenged monolayers compared with unchallenged monolayers (Fig. 1E and F). Live *T. gondii* tachyzoites, but not multiplicity of infection (MOI)-equivalent amounts of tachyzoite lysate, selectively induced elevated *Timp1*, but not *Timp2*, expression (Fig. 1G). In a similar fashion, Caco-2 cell monolayers responded to challenge with live *T. gondii* tachyzoites with increased *TIMP1*, but not *TIMP2*, mRNA expression (Fig. 1H) and with TIMP1 protein secretion in supernatants (Fig. 1I), contrasting with the non-significant effects of stimulation with LPS or tachyzoite lysates (Fig. 1H). We conclude that *T. gondii* infection elevates mRNA expression and protein secretion of TIMP1 by murine brain endothelial cell and human gut epithelial cell monolayers.

### Effects of recombinant TIMP1 on parasite transmigration across polarized cell monolayers *in vitro*

**Fig 2:**
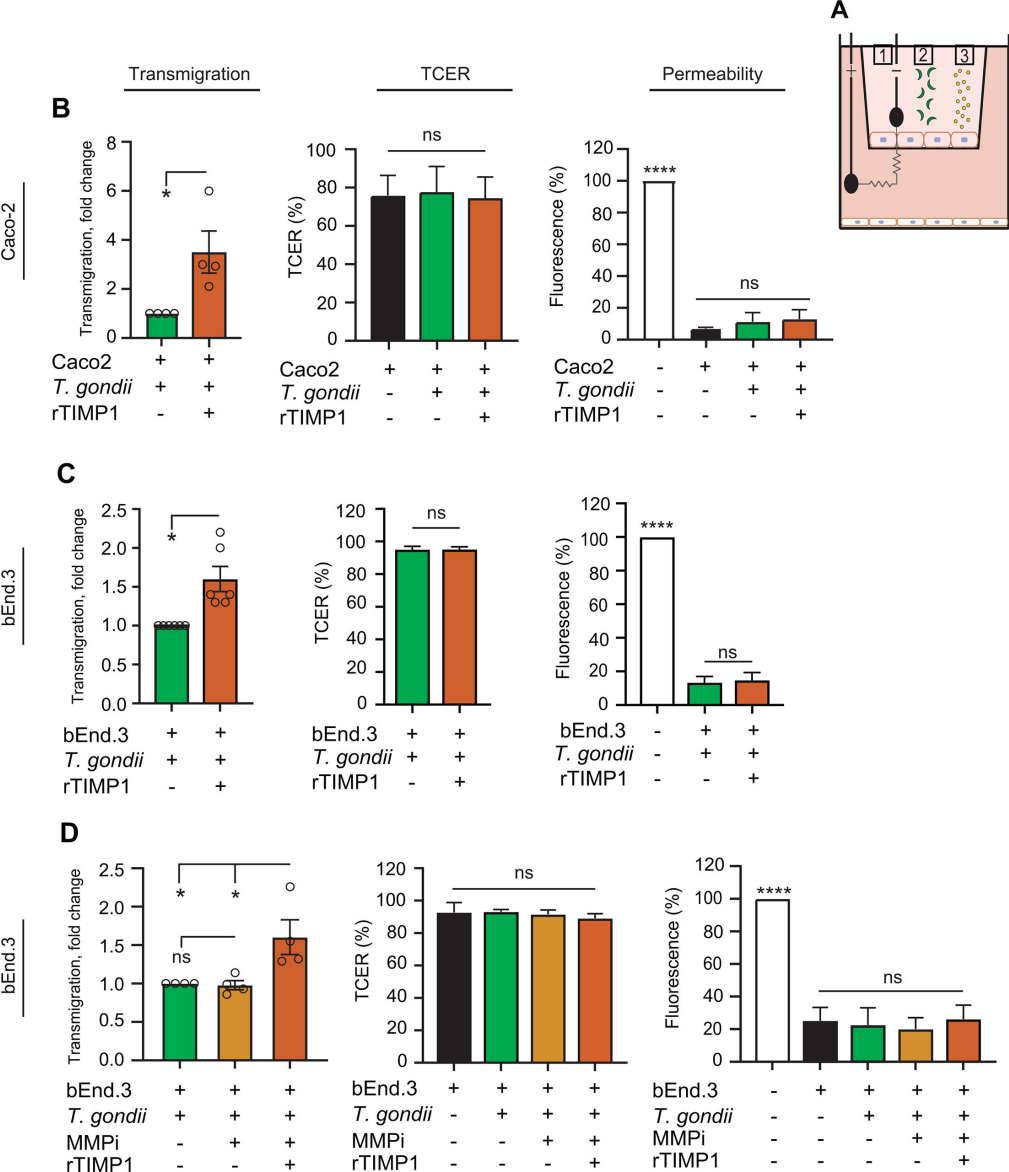
Transmigration of *T. gondii* across polarized cell monolayers in presence of recombinant TIMP1 (rTIMP1) protein and MMP inhibition. (**A**) Schematic representation of experimental setup in a transwell filter system with polarized cellular monolayer in the upper well and HFF monolayer in the lower well. (1) TCER measurement, (2) freshly egressed *T. gondii* tachyzoites added to upper well, and (3) FITC-dextran (3 kDa) fluorescent permeability tracer added to the upper well and measured in the lower well. (**B**) Transmigration of *T. gondii* tachyzoites (RH-LDM) across polarized Caco-2 cell monolayers, TCER, and permeability to FITC-dextran of cell monolayers. Where indicated, rTIMP1 (100 ng/mL) was added to the upper compartment of the transwells together with tachyzoites. (**C**) Transmigration of *T. gondii* tachyzoites (PRU A7) across polarized bEnd.3 cell monolayers, TCER, and permeability to FITC-dextran of cell monolayers. Where indicated, rTIMP1 (100 ng/mL) was added to the upper compartment of the transwells together with tachyzoites. (**D**) Transmigration of *T. gondii* tachyzoites (PRU A7) across polarized bEnd.3 cell monolayers, TCER, and permeability to FITC-dextran of cell monolayers. Where indicated, cell monolayers were pretreated with MMP inhibitor GM6001 (MMPi, 25 µM), and rTIMP1 (100 ng/mL) was added to the upper compartment of the transwells together with tachyzoites. Transmigration is displayed as fold change in relation to transmigration of *T. gondii* tachyzoites across mock-treated cell monolayers. TCER is shown as % TCER values (Ω • cm^2^) relative to TCER values at the initiation of the assay (100%), as detailed in Materials and Methods. Permeability to FITC-dextran was measured as arbitrary fluorescence units (AU) from leaked FITC-dextran in the lower transwell chamber at the end of the transmigration assay and related to the signal of the transwell insert in the absence of a polarized monolayer (100%). Data are presented as mean (±SEM) from three to six independent experiments per condition. Statistical analyses were performed with Student’s *t*-test for two sample comparisons and one-way analysis of variance followed by Sidak post hoc test for multiple comparisons, *P < 0.05, ****P < 0.0001, ns: non-significant.

Given that TIMP1 has been associated with protective roles in maintaining barrier integrity in BBB and intestinal barrier models (31, 32), we investigated the potential impact of recombinant TIMP1 (rTIMP1) on parasite transmigration and cellular barrier integrity in transwell assays (11) (Fig. 2A). Unexpectedly, in the presence of rTIMP1, the transmigration frequencies of *T. gondii* tachyzoites (type I) across polarized Caco-2 monolayers were significantly increased, with maintained transcellular electrical resistance (TCER) values and impermeability to FITC-dextran (Fig. 2B). These results were confirmed using polarized bEnd.3 monolayers and type II tachyzoites (Fig. 2C). Finally, because TIMP1 inhibits the activity of MMPs, we assessed if broad MMP inhibition impacted transmigration of tachyzoites. Contrasting with previously reported impacts on the transmigration of *T. gondii*-infected dendritic cells (33, 34), MMP inhibition non-significantly impacted the transmigration frequencies of tachyzoites (Fig. 2D). Further, addition of rTIMP1 in the presence of MMP inhibitor elevated transmigration, corroborating previous results and indicative of MMP-independent effects of TIMP1 on transmigration (Fig. 2D).

Next, we attempted targeting *Timp1* with shRNA-mediated gene silencing (34) (Fig. S1A and B). *Timp1* expression was not abolished, albeit significantly reduced in shTIMP1-transduced bEnd.3 cells live-sorted after transduction (Fig. S1C). We confirmed that secretion of TIMP1 was reduced by shTIMP1 treatments compared with vector control condition (shLuc) (Fig. S1D). However, TIMP1 secretion re-elevated upon challenge with *T. gondii* (Fig. S1D), indicating maintained responsiveness despite shTIMP1. As would be anticipated from the BBB stabilizing roles attributed to TIMP1, *Timp1* silencing consistently elevated the transmigration frequencies of *T. gondii* tachyzoites, in the absence of measurable impacts on the TCER and permeability of monolayers (Fig. S1E through G). Jointly, the elevation of transmigration frequencies by *T. gondii* tachyzoites in presence of rTIMP1 or upon *Timp1* gene silencing motivated a further exploration.

### Host cell signaling pathways and parasite secretory pathways with an impact on *Timp1* expression

**Fig 3:**
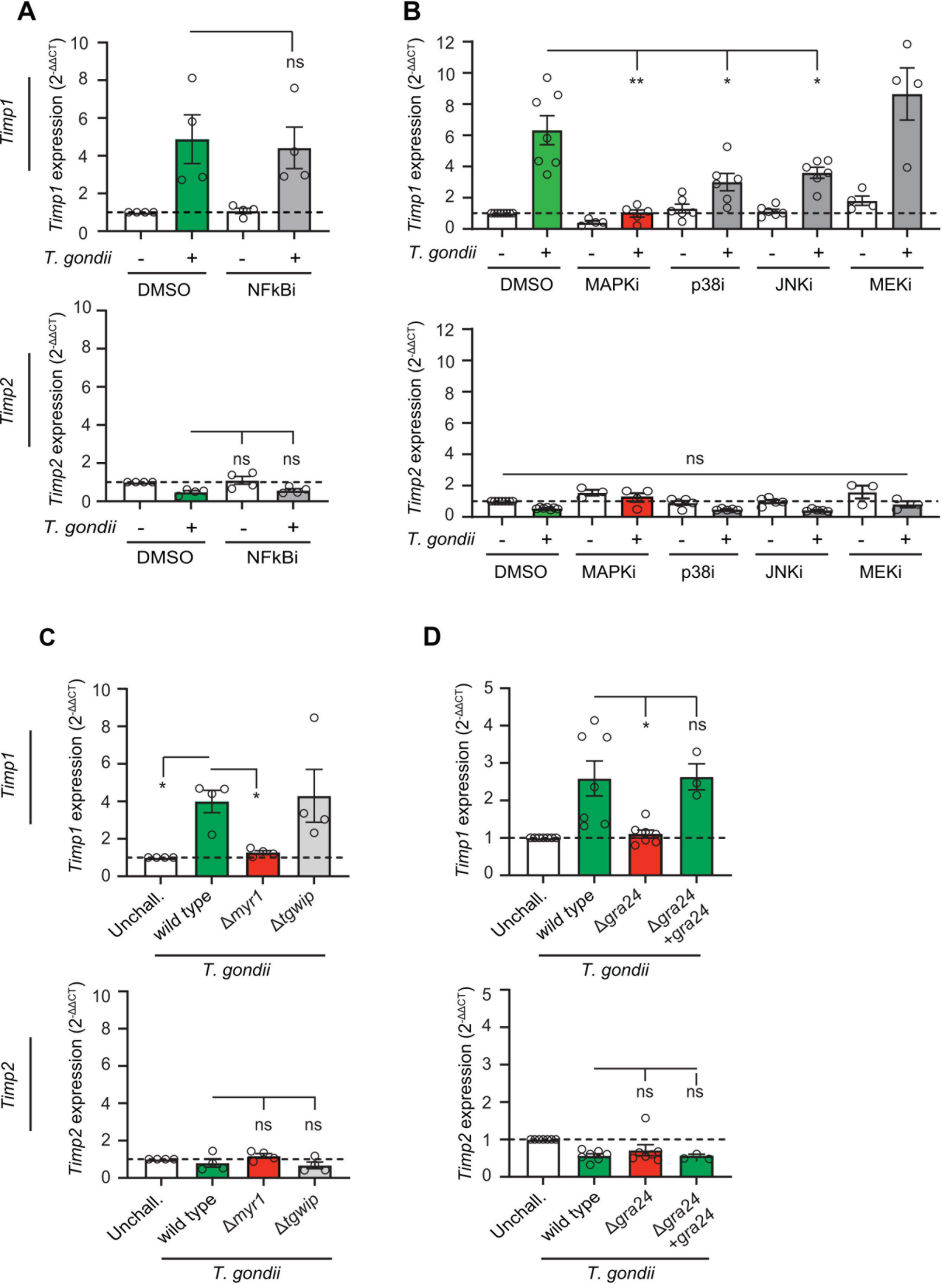
*Timp1* expression upon challenge with effector MYR1 and GRA24 *T. gondii* mutants and inhibition of cell signaling pathways. (**A**) qPCR analyses of *Timp1* and *Timp2* cDNA from bEnd.3 cells challenged with *T. gondii* tachyzoites (RH-LDM, MOI 2) for 24 h in presence of NFkB/STAT3 inhibitors JSH-23 (9 µM) and TPCA-1 (3 µM) or DMSO as a vehicle control. (**B**) qPCR analyses of *Timp1* and *Timp2* cDNA from bEnd.3 cells challenged with *T. gondii* tachyzoites (RH-LDM, MOI 2) for 24 h in presence of p38 MAP kinase inhibitor birb 796 (p38i, 5 µM), MEK inhibitor trametenib (MEKi, 5 µM), JNK-IN-8 (JNKi, 1 µM), combination of all three (MAPKi), or DMSO as a vehicle control. (**C**) qPCR analyses of *Timp1* and *Timp2* cDNA from bEnd.3 cells challenged with *T. gondii* tachyzoites RH1-1 (parental wild type), MYR1-deficient mutant (Δ*myr1*), and TgWIP-deficient mutant (Δ*tgwip*) for 24 h with MOI 2. (**D**) qPCR analyses of *Timp1* and *Timp2* cDNA from bEnd.3 cells challenged with *T. gondii* tachyzoites RH Ku80 (parental wild type), GRA24-deficient mutant (Δ*gra24*), and reconstituted mutant (Δ*gra24* + gra24) for 24 h with MOI 2. Data are presented as mean (±SEM) from three to seven independent experiments per condition. qPCR data are displayed as fold change (2^−ΔΔCt^) in relation to unchallenged condition. Statistical comparisons were performed with repeated measures analysis of variance, Sidak post hoc test, *P < 0.05, **P < 0.01, ns: non-significant.

To investigate the cell signaling that implicated the upregulated expression of *Timp1* upon *T. gondii* infection, we first applied pharmacological inhibition targeting pathways associated with TIMP1 regulation in other systems (35–37). Inhibitors of NF-kB and STAT signaling, separately or in combination, non-significantly impacted *Timp1* expression in *T. gondii*-challenged cells (Fig. 3A; Fig. S2A). In sharp contrast, inhibitors of p38 and JNK MAP kinases, individually or in combination, reduced or abolished, respectively, *Timp1* upregulation upon *T. gondii* challenge (Fig. 3B), while MEK (ERK1/2) inhibition non-significantly impacted *Timp1* expression (Fig. 3B). Guided by these pharmacological inhibition data, we next investigated the putative involvement of *T. gondii* effectors in a loss-of-function screen using various *T. gondii* mutants. Initially, we assessed MYR1 due to its role in the translocation of multiple effectors from the parasitophorous vacuole into the host cell cytosol (16) and the dependence of MYR1 responses on JNK MAPK (38). Interestingly, depletion of MYR1 selectively abolished the elevated expression of *Timp1*, with a non-significant impact on *Timp2* expression (Fig. 3C). Consistent with this, depletion of the effector GRA24, which is translocated via MYR (16) and can act via p38 MAPK (39), abolished *Timp1* induction and reconstitution of GRA24 in mutant re-elevated *Timp1* expression (Fig. 3D). In contrast, *Timp1* expression was undiminished for a set of MYR-associated and MYR-unassociated effector mutants linked to various other signalling pathways of the host cell (TgWIP, GRA15, ROP16, TgIST, and TEEGR) (Fig. 3C; Fig. S2B). Overall, non-significant effects were measured on *Timp2* expression (Fig. 3A through D; Fig. S2C), indicating selective effects on *Timp1*. Altogether, these data identify the implication of MYR1-dependent GRA24 effector in the elevated expression of *Timp1*.

### Roles of p38 MAP kinase signaling and parasite effector GRA24 in *Timp1* expression and tachyzoite transmigration

**Fig 4:**
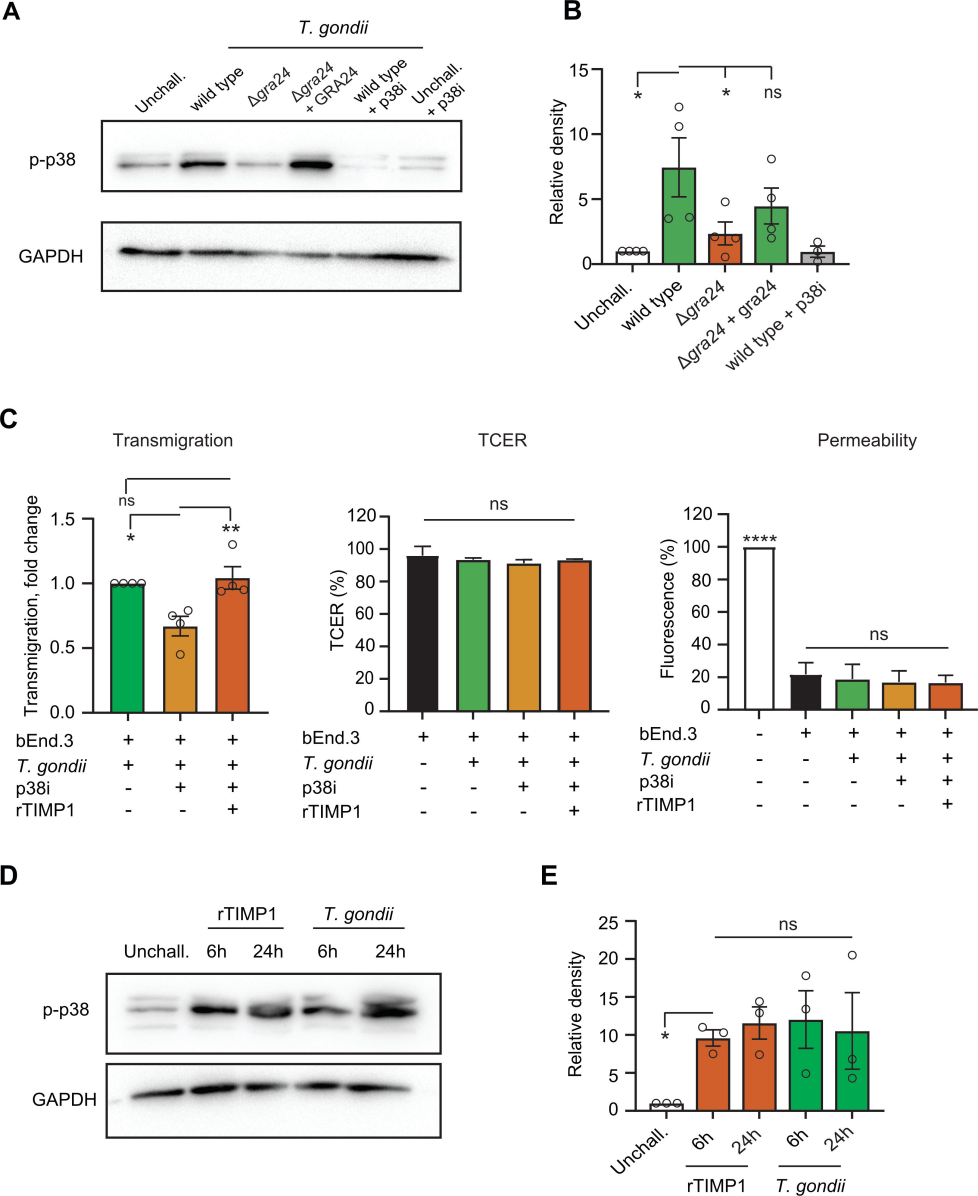
Phosphorylation of p38 MAPK in bEnd.3 cells upon rTIMP1 treatment or *T. gondii* challenge and its impact on *T. gondii* transmigration. (**A and B**) Western blot analysis of phospho-p38 (Thr180/Tyr182) levels in bEnd.3 cells challenged with *T. gondii* tachyzoites RH Ku80 (parental wild type), GRA24-deficient mutant (Δ*gra24*) and reconstituted mutant (Δ*gra24* + gra24) for 24 h with MOI 2 in presence or absence of p38 MAP kinase inhibitor birb 796 (p38i, 5 µM). Bar graph (**B**) displays the relative density of phospho-p38 signal relative to GAPDH signal. (**C**) Transmigration of *T. gondii* tachyzoites (PRU A7) across polarized bEnd.3 cell monolayers, TCER, and permeability to FITC-dextran (3 kDa) of cell monolayers. Where indicated, cell monolayers were pretreated with p38 MAP kinase inhibitor birb 796 (p38i, 5 µM), and rTIMP1 (100 ng/mL) was added to the upper compartment of the transwells together with tachyzoites. Transmigration is displayed as fold change in relation to transmigration of *T. gondii* tachyzoites across mock-treated cell monolayers. TCER is shown as % TCER values (Ω • cm^2^) relative to TCER values at the initiation of the assay (100%), as detailed in Materials and Methods. Permeability to FITC-dextran was measured as arbitrary fluorescence units (AU) from leaked FITC-dextran in the lower transwell chamber at the end of the transmigration assay and related to the signal of the transwell insert in the absence of a polarized monolayer (100%). (**D and E **) Western blot analysis of phospho-p38 (Thr180/Tyr182) levels in bEnd.3 cells challenged with *T. gondii* tachyzoites (RH LDM, MOI 4 for 6 h and MOI 2 for 24 h) or treated with recombinant TIMP1 protein (100 ng/mL) for 6 h and 24 h. Bar graphs (**E**) display the relative density of phospho-p38 signal relative to GAPDH signal. Data are presented as mean (±SEM) from three to four independent experiments. Statistical comparisons were performed with one-way analysis of variance, Sidak post hoc test, *P < 0.05, **P < 0.01, ****P < 0.0001, ns: non-significant.

To confirm the impact of GRA24 on p38 MAP kinase signaling in polarized endothelium, bEnd.3 monolayers were challenged with *T. gondii* GRA24 mutants or p38 inhibition and assessed by Western blotting. The data consistently showed GRA24-dependent p38 phosphorylation in bEnd.3 cells, which was abolished upon pharmacological inhibition of p38 (Fig. 4A and B), aligning with findings in other cell types (39). Importantly, p38 inhibition reduced the transmigration frequency of tachyzoites, and addition of rTIMP1 re-elevated transmigration in the presence of the p38 inhibitor (Fig. 4C), confirming the pro-transmigratory impact of TIMP1. Finally, taking into consideration that MAP kinase signalling can be cross-regulated, we investigated the effect of TIMP1—secreted upon *T. gondii* infection—on p38 activation. We found that rTIMP1 consistently elevated p38 phosphorylation, similar to the response seen during *T. gondii* challenge (Fig. 4D). This suggests that TIMP1 secreted by infected cells may also activate p38 signalling in non-infected bystander cells. Jointly, the data show an implication of the parasite effector GRA24 and host p38 MAPK signaling in the elevated expression of *Timp1* and in the transmigration of *T. gondii* tachyzoites across polarized monolayers.

## DISCUSSION

In this study, we identify a role for TIMP1 in facilitating the translocation of *T. gondii* across cellular barriers formed by polarized endothelial and epithelial monolayers *in vitro*. We observed that exposure to *T. gondii* tachyzoites, but not to parasite lysates or LPS stimulation, significantly increased *Timp1* mRNA expression and TIMP1 secretion in cell supernatants. In contrast, *Timp2* mRNA expression remained largely unchanged in both cell types. Our findings show that recombinant TIMP1 enhances the transmigration of *T. gondii* tachyzoites across polarized monolayers. Interestingly, this increased transmigration occurred without measurable changes in TCER, permeability to FITC-dextran, or disruption of TJs (ZO-1). This suggests that TIMP1 increases the permissiveness of the monolayers to *T. gondii* passage while preserving overall barrier integrity. The observed TIMP1-related increase in *T. gondii* transmigration prompted further investigation into the signaling pathways involved in *Timp1* expression.

We demonstrate that the parasite effector GRA24, secreted through the MYR translocon, induces elevated *Timp1* expression via p38 MAPK signaling. Pharmacological inhibition of the major MAPK pathways, specifically p38 and JNK (but not Erk), significantly reduced *Timp1* expression. These findings align with previous studies on JNK-dependent MYR1 responses (38) and p38 MAPK-dependent GRA24 effects (39). Additionally, while TIMP1 expression has been linked to NF-kB signaling in malignancies (37), neither pharmacological inhibition of NF-kB nor the use of a *T. gondii* line deficient in GRA15, a known NF-kB activator (40), had a measurable impact on *Timp1* expression compared to wild type. GRA15 and GRA24 proteins cooperate in the induction of pro-inflammatory and pro-migratory responses, for example, the induction of IL-12 responses in peripheral blood mononuclear cells (41) or pro-migratory signaling in infected phagocytes (42). Yet, the data indicate that GRA24 (p38), and not GRA15 (NF-kB), is the main driver of TIMP1 expression in epithelial or endothelial cells. Similarly, since STAT activation is associated with TIMP1 expression (43), we tested a *T. gondii* mutant lacking ROP16, a STAT-activating effector (44), and found no impact on *Timp1* expression. Collectively, the data indicate that GRA24/p38 MAPK signaling, rather than GRA15/NF-kB or ROP16/STAT signaling, is the primary pathway driving *T. gondii*-induced *Timp1* expression in cellular monolayers.

We report that recombinant TIMP1 increases the transmigration frequency of *T. gondii* tachyzoites across polarized monolayers, suggesting a direct influence on the translocation process. This consistent effect occurred independently of MMP inhibition and was similarly present upon challenge with types I and II *T. gondii* strains, despite the inherent differences in transmigration frequencies between these two *T. gondii* genotypes (9) and the inherent differences in resistance to transmigration that Caco-2 and bEnd.3 polarized monolayers offer (11). Further, bEnd.3 endothelial cells exhibited resilience to *Timp1* gene silencing compared with dendritic cells (34). Nonetheless, upon significant *Timp1* gene silencing, transmigration remained elevated, with the ultrastructure (ZO-1) and impermeability of the cellular barriers intact. Thus, both the addition of recombinant TIMP1 and *Timp1* gene silencing appeared to favor *T. gondii* transmigration. These seemingly contradictory results may reflect a transient destabilization of barrier function in both scenarios, likely linked to TIMP1’s pleiotropic and MMP-independent roles. Similarly, in cancer, TIMP1 is known to inhibit tumor progression as a natural MMP inhibitor (45). Yet, overexpression of TIMP1 by various cancer types has been associated with increased disease severity and pro-metastatic effects (25), partly mediated by TIMP1/CD63/ITGB1 activation of focal adhesion kinase (FAK) signaling (46). These findings illustrate how an improved understanding of TIMP1’s dual roles as both an MMP inhibitor and signaling molecule helps to reconcile previously contradictory observations (27). Because FAK is dysregulated upon *T. gondii* infection (11), future investigations need to address if *T. gondii*-induced TIMP1 impacts FAK and consequently TJ stability. In other settings, TIMP1 was reported to influence the dephosphorylation of FAK (*Ptk2*) and endothelial cell motility (47, 48). In *T. gondii*-challenged endothelial and Caco-2 cells, FAK dephosphorylation occurs alongside transient TJ destabilization, promoting increased *T. gondii* transmigration (11). On the other hand, TIMP1 has also been linked to protective roles at the BBB, primarily through MMP inhibition (31) but also via interactions with CD63/ITGB1 (49), which help preserve TJs (50). Similarly, in intestinal models, TIMP1 has been shown to maintain TJ integrity (e.g., occludin) in Caco-2 monolayers (32), likely due to its MMP-inhibitory function (51, 52). In addition, the intriguing finding that rTIMP1 can trigger phosphorylation of p38 MAK kinase indicates that non-infected bystander endothelial cells also may contribute to facilitating transmigration, and this warrants further investigation. Together, the present findings of GRA24-induced TIMP1 signalling add to the pro-transmigratory effects of *T. gondii* infection on polarized cellular monolayers via FAK signaling (15, 53) and, jointly, likely play crucial roles in facilitating the translocation of *T. gondii* tachyzoites across polarized monolayers.

Our findings suggest a pathophysiological role for TIMP1 in *T. gondii* infection. Elevated TIMP1 levels have been detected in the serum and brain tissue of *T. gondii*-infected mice (15, 29), with reduced cerebral parasitic loads measured in TIMP1-deficient mice (30). Moreover, *T. gondii*-infected dendritic cells secrete TIMP1, affecting their motility and transmigration (33, 34). Thus, elevated TIMP1 levels in the vicinity of invasive parasite clusters in the tissue microenvironment, that is, specifically around the cerebral microvasculature (14, 15), may promote parasite dissemination while inhibiting MMP-mediated inflammation. We speculate that TIMP1 elevation exerts a pro-disseminatory effect by facilitating the transmigration of both extracellular tachyzoites and infected leukocytes (*Trojan horse* mechanism). Simultaneously, reduced proteolysis (33) may locally dampen inflammation in the intestinal epithelium or cerebral perivascular environment during toxoplasmosis. Supporting this idea, the intestinal phase of toxoplasmosis is typically clinically silent in humans (7) and shows minimal tissue inflammation in rodent models (54, 55). Similarly, colonization of the CNS during primary natural infection in humans and mice is generally asymptomatic or accompanied by mild symptoms and occurs in the absence of focal neurological symptoms (7), aligning with recent findings that suggest very early *T. gondii* passage to the CNS (15, 53).

Bacterial and viral infections can disrupt the balance of MMP/TIMP regulation (23). Because *T. gondii* infection elevates TIMP1 expression, but to a lesser extent MMP expression *in vitro*, it is plausible that the anti-inflammatory effects of TIMP1 prevail, as indicated by decreased proteolysis *in vitro* (33). However, whether this pattern extends to *in vivo* infections remains to be investigated. Elevated TIMP1 levels have also been reported in serum and cerebrospinal fluid during severe *Plasmodium falciparum* malaria (56) and during viral or bacterial meningitis (57, 58). Finally, while TIMP1 might be considered a biomarker for inflammatory and CNS injuries, its elevation is not a consistent feature. For instance, in contrast to our findings, TIMP2—not TIMP1—was elevated in cellular models and human brain biopsies of *Mycobacterium tuberculosis* infection (59). More recently, TIMP2 has also been linked to protective effects on the BBB (60).

In summary, the present data reveal a role for TIMP1 in the translocation of *T. gondii* across polarized endothelial and epithelial cell linings. Based on the current *in vitro* findings, we postulate that TIMP1 facilitates *T. gondii* dissemination by (i) promoting the transmigration of extracellular tachyzoites, (ii) enhancing the dissemination of parasitized leukocytes (33, 34), and (iii) reducing proteolytic MMP-related effects, which likely dampens inflammation. Thus, the specific roles of TIMP1 identified here warrant further in-depth investigation *in vivo*.

## MATERIALS AND METHODS

### Cell lines, parasite culture, and infection challenges

Human foreskin fibroblasts (HFF-1 SCRC-1041, American Type Culture Collection), Caco-2 cells (HTB-37, American Type Culture Collection), and bEnd.3 cells (CRL-2299, American Type Culture Collection) were cultured in Dulbecco’s modified Eagle’s medium, high glucose (HyClone), with 10% fetal bovine serum (FBS; HyClone), 20 µg/mL gentamicin (Sigma-Aldrich), and 2 mM L-glutamine, referred to as DMEM. All cell cultures used were periodically tested for mycoplasma and found to be negative.

*T. gondii* tachyzoites were maintained by serial 48-h passages in HFF-1 monolayers. *T. gondii* lines and reagents used are listed in Table S1. Caco-2 and bEnd.3 cells were challenged with freshly egressed *T. gondii* tachyzoites to reach the infection frequency of 50–80%. When indicated, cells were treated with inhibitors 2 h prior to challenge, lipopolysaccharide (LPS; Sigma-Aldrich) or *T. gondii* lysate, generated from freshly egressed *T. gondii* tachyzoites by repeated freeze-thaw cycles.

### Cell polarization and transmigration assay

Caco-2 or bEnd.3 cells were cultured to 80% confluence and then seeded onto culture inserts (8-µm pore size; Corning Transwell or cellQART Sabeu) and grown for 5 days until they reached polarization, defined as a stable TCER for 2 days above 250 Ω • cm^2^ (Caco-2) and 180 Ω • cm^2^ (bEnd.3). TCER was measured before and after transmigration using an Ohmmeter (Millipore, Bedford, MA) and measurements calculated with the following formula: unit area resistance (TCER) = resistance (Ω) • effective membrane area (cm^2^). Values are shown as percentages of TCER related to TCER prior to transmigration. For evaluation of cell monolayer permeability following transmigration, FITC-dextran (3 kDa; Life tech) was added to the upper compartment of the culture insert at a concentration of 12.5 µg/mL for 90 min. The medium was collected from the lower compartment, and fluorescence was measured in a fluorometer (EnSpire Multimode Plate Reader, Perkin Elmer) at 485-nm excitation and 520-nm emission.

Transmigration assay was performed as previously described (9), with modifications. Briefly, freshly egressed tachyzoites were transferred to transwell inserts with polarised monolayers of Caco-2 or bEnd.3 cells and allowed to transmigrate for 18 h. When indicated, rTIMP1 was added to the cells at a concentration of 100 ng/mL together with tachyzoites. After 24–36 h, transmigrated tachyzoites were quantified by plaquing assays. GFP^+^ intracellular vacuoles with replicating tachyzoites in the underlying HFF monolayers were counted by epifluorescence microscopy (1 vacuole = 1 transmigrated tachyzoite).

### Immunofluorescence microscopy

Caco-2 and bEnd.3 cells were seeded on gelatin (1%)-coated glass coverslips, challenged with freshly egressed *T. gondii* tachyzoites (RH LDM; MOI 2) for 6 h or left unchallenged and fixed with 4% paraformaldehyde (PFA). Cells were then permeabilized with 0,1% Triton X-100 in PBS and stained with primary anti-ZO1 and anti-rabbit IgG Alexa Fluor 594 (Thermo Scientific) secondary antibodies and DAPI. Images were acquired on a Leica DMi8 with a 63× objective or Zeiss Z1 Observer with a 20× objective.

### Lentiviral vector production and *in vitro* transduction

Self-inactivating lentiviral vector pLL3.7 (11,795, Addgene) was used to express shRNA targeting mouse *Timp-1* mRNA (shTIMP1) and a non-related sequence (luciferase, shLuc). Self-complementary hairpin DNA oligos were chemically synthesized (Eurofins Genomics, Sweden), aligned, and ligated in pLL3.7, containing a CMV-driven eGFP (shTIMP1 and shLuc) reporter and a U6 promoter upstream of the cloning restriction sites (HpaI and XhoI). Transfer plasmid (shTIMP1 or shLuc) was co-transfected with psPAX2 (12260, Addgene) packaging vector and pCMV-VSVg (8454, Addgene) envelope vector into Lenti-X 293T cells (Clontech) using Lipofectamine 2000 (Invitrogen). The resulting supernatant was harvested 24 and 48 h post-transfection. Recovered lentiviral particles were centrifuged to eliminate cell debris and filtered through 0.45mm cellulose acetate filters.

bEnd.3 cells were transduced by incubation with lentiviral particles in the presence of diethylaminoethyl dextran (DEAE-dextran, 8 µg/mL; Sigma). Five days post-transduction, cells were subcultured and grown to 80% confluence. Then cells were trypsinized, washed with FACS buffer (2% FBS, 2 mM EDTA in PBS), subjected to fluorescence-activated cell sorting (BD FACSMelody), and transferred to culture flasks to generate stable lines (Fig. S1). Mock-treated bEnd.3 cells were subjected to the same procedure, but an eGFP-negative population was collected. eGFP expression was verified by epifluorescence microscopy before cells were used in experiments.

### Quantitative polymerase chain reaction

Cells were cultured in DMEM or challenged as indicated and lysed in TRI Reagent (Sigma-Aldrich) or lysis buffer (Jena Bioscience). Total RNA was extracted according to the manufacturer’s protocol using the Direct-zol RNA Miniprep (Zymo Research) or Total RNA Purification Kit (Jena Bioscience) and reverse-transcribed with Maxima H Minus Reverse Transcriptase (Thermo Fisher). Real-time quantitative polymerase chain reaction (qPCR) was performed with SYBR green PCR master mix (KAPA biosystems) or HotStart 2× SYBR Green qPCR Master Mix (APExBio Technology), specific forward and reverse primers at target-dependent concentrations (100–200 nM), and cDNA (10–15 ng) in a QuantStudio 5 System (Thermo Fisher) with ROX as a passive reference. qPCR results were analyzed using the ΔCt method relative to importin-8 and TATA-binding protein as housekeeping genes and displayed as fold change to unchallenged (set to 1). Primer sequences are listed in Table S1.

### Western blot

BEnd.3 cell monolayers were grown on 12-well plates and treated or challenged with *T. gondii* as stated. Cells were lysed with radioimmunoprecipitation assay (RIPA) buffer (150 mM NaCl, 0.1% Triton X-100, 0.5% sodium deoxycholate, 0.1% sodium dodecyl sulfate [SDS], 50 mM Tris-HCl, pH 8.0, protease inhibitors, and phosphatase inhibitors) on ice and then mixed with Laemmli buffer and boiled at 95°C for 5 min. Proteins were separated on 10% SDS-PAGE gel and blotted onto polyvinylidene fluoride (PVDF) membrane, blocked in 5% BSA followed by incubation with primary and secondary antibodies: anti-phospho-p38 (Cell Signaling), anti-glyceraldehyde-3-phosphate dehydrogenase (anti-GAPDH; Millipore), and anti-rabbit IgG-HRP (Cell Signaling). Proteins were revealed by enhanced chemiluminescence in a BioRad ChemiDoc XRS+ (GE Healthcare). Densitometry analysis was performed using ImageLab version 6.1.0 (Bio-Rad Laboratories, Inc.).

### TIMP1 ELISA

Supernatants from Caco-2 and bEnd.3 cells challenged with freshly egressed *T. gondii* tachyzoites (RH LDM) were collected and analyzed using ELISA (Human TIMP-1 Quantikine ELISA Kit or Mouse TIMP-1 Quantikine ELISA Kit, R&D Systems) according to the manufacturer’s instructions.

### Statistical analyses

Statistical analyses were performed with Prism software (GraphPad v. 9) or with R version 4.2.3 and RStudio 2021.09.2+382. Hypothesis tests used are indicated in the figure legends and were chosen based on experimental design, the hypothesis to be tested, data distribution, and which statistics were to be presented. The area under the curve was calculated with the R package PKNCA. In all tests, statistical significance is defined as *P* < 0.05.

## Data Availability

The data sets generated during and/or analyzed during the current study are available from the corresponding author on reasonable request.

## SUPPLEMENTAL MATERIAL

Supplemental material msphere.00102-25-s0001.pdf Table S1; Figures S1 and S2.

ASM does not own the copyrights to Supplemental Material that may be linked to, or accessed through, an article. The authors have granted ASM a non-exclusive, world-wide license to publish the Supplemental Material files. Please contact the corresponding author directly for reuse.
