# Supplementary material for: TIMP1 secretion induced by Toxoplasma effector GRA24 via p38 MAPK signaling promotes non-disruptive parasite translocation across polarized brain endothelial monolayers
Source: mSphere. 2025 Apr 23;10(5):e00102-25. doi: 10.1128/msphere.00102-25 (PMC12108053; doi:10.1128/msphere.00102-25)
Supplement: Supplemental material — Table S1; Figures S1 and S2. [file msphere.00102-25-s0001.pdf]

Supplementary Material

TIMP1 secretion induced by Toxoplasma effector GRA24 via p38 MAPK signaling promotes non-disruptive parasite translocation across polarized brain endothelial monolayers

Elena Afanaseva[1], Antonio Barragan[1]

[1]Department of Molecular Biosciences, The Wenner-Gren Institute, Stockholm University, Stockholm, Sweden

Table S1
Figure S1
Figure S2

Table S1. Key resources used in the study

REAGENT or RESOURCE	SOURCE	IDENTIFIER
**Antibodies**		
Anti-ZO-1 (Rabbit polyclonal)	Thermo Fisher	Cat# 40–2200, RRID:AB_2533456
Anti-Rabbit IgG (H + L) Alexa Fluor 594 (Chicken polyclonal)	Thermo Fisher	Cat# A-21442, RRID:AB_2535860
Anti-phospho-p38 MAPK (Thr180/Tyr182) (D3F9)	Cell signaling	Cat# 4511
Anti-GAPDH	Millipore	ABS16
Anti-rabbit IgG, HPR-linked	Cell signaling	#7074
**Chemicals and recombinant proteins**		
Parthenolide NF-κB inhibitor	MedChemExpress	Cat# HY-N0141
TPCA-1 IKK-2 and STAT3 inhibitor	MedChemExpress	Cat# HY-10074
JSH-23 p65 inhibitor	MedChemExpress	Cat# HY-13982
BIRB 796 p38 MAPK inhibitor	MedChemExpress	Cat# HY-10320
Trametinib MEK1 and MEK2 inhibitor	MedChemExpress	Cat# HY-10999
JNK-IN-8 JNK inhibitor	MedChemExpress	Cat# HY-13319
Src inhibitor 1	MedChemExpress	Cat# HY-101053
MK2206 Akt inhibitor	MedChemExpress	Cat# HY-108232
GM6001 MMP inhibitor	MedChemExpress	Cat# HY-15768
Dextran Fluorescein 3000 MW anionic	Thermo Fisher	Cat# D3305
DEAE-dextran	Sigma Aldrich	
Recombinant Human TIMP-1 Protein CF	R&D Systems	Cat# 970-TM
CellTracker Orange CMTMR Dye	Thermo Fisher	Cat# C2927
Lipopolysaccharides from *Escherichia coli* O111:B4	Sigma Aldrich	Cat# L2630
**Commercial assays**		
Human TIMP-1 Quantkine ELISA kit	R&D Systems	Cat # DTM100
Mouse TIMP-1 Quantkine ELISA kit	R&D Systems	Cat # MTM100
Direct-zol RNA Miniprep kit	Zymo Research	Cat# R2052
Total RNA purification kit	Jena Bioscience	Cat# PP-210L
**Cell lines**		
Human foreskin fibroblasts HFF-1	American Type Culture Collection	Cat# SCRC-1041 RRID:CVCL_3285
Caco-2	American Type Culture Collection	Cat# ATCC HTB-37 RRID:CVCL_0025
bEnd.3	American Type Culture Collection	Cat# ATCC CRL-2299 RRID:CVCL_0170
**Parasite strains**		
T. gondii RH-LDM GFPS65T	(Barragan and Sibley, 2002; Kim et al., 2001)	N/A
T. gondii RH1-1 cLuc GFP^+	(Boyle et al., 2007)	N/A
T. gondii RH Δ*myr1* cLuc GFP^+	(Wang et al., 2019)	N/A
T. gondii RH Δ*tgwip* GFP^+	(Sangare et al., 2019)	N/A
T. gondii RH Ku80	(Huynh and Carruthers, 2009)	N/A

T. gondii RH Ku80 Δ*gra24*	(Braun et al., 2013)	N/A
T. gondii RH Ku80 Δ*gra24* + *gra24*	(Braun et al., 2013)	N/A
T. gondii Pru Δ*hpt* GFP^+ (PRU A7)	(Kim et al., 2007)	N/A
T. gondii Pru Δ*hpt* GFP^+ Δ*gra15*	(Mukhopadhyay et al., 2020)	N/A
T. gondii RH Δ*hpt*	(Jensen et al., 2011)	N/A
T. gondii RH Δ*hpt* Δ*rop16*	(Jensen et al., 2011)	N/A
T. gondii Pru Δ*ku80*	(Braun et al., 2019)	N/A
T. gondii Pru Δ*ku80* Δ*teegr*	(Braun et al., 2019)	N/A
T. gondii Pru Δ*ku80* Δ*tgist*	(Gay et al., 2016)	N/A
**Software and algorithms**		
ImageJ	(Schneider et al., 2012)	https://imagej.nih.gov/ij/
GraphPad Prism 9.0	GraphPad Software	http://www.graphpad.com
FlowJo X 10.9.0	Flowjo LLC	http://www.flowjo.com
**qPCR primers**		
Target	Sequence 5'-3'	
mouse *Timp1* fw	GCAACTCGGACCTGGTCATAA	
mouse *Timp1* rv	CGCTGGTATAAGGTGGTCTCG	
mouse *Timp2* fw	CTCGCTGTCCCATGATCCC	
mouse *Timp2* rv	GCCCATTGATGCTCTTCTCTGT	
mouse *Ipo8* fw	CTATGCTCTCGTTCAGTATGC	
mouse *Ipo8* rv	GTCCGAAAGATCTCCATCCA	
mouse *Tbp* fw	GGGGAGCTGTGATGTGAAGT	
mouse *Tbp* rv	CCAGGAAATAATTCTGGCTCA	
human *TIMP1* fw	AGACCTACACTGTTGGCTGTGAG	
human *TIMP1* rv	GACTGGAAGCCCTTTTCAGAG	
human *TIMP2* fw	ATGCACATCACCCTCTGTGA	
human *TIMP2* rv	CTCTGTGACCCAGTCCATCC	
human *IPO8* fw	CGAAGCTCACTAGTTTTGACCC	
human *IPO8* rv	GCAAAGGAAGGGGAATTGA	
human *TBP* fw	TCTGGGTTTGATCATTCTGTAG	
human *TBP* rv	GAGCTGTGATGTGAAGTTTCC	
**shRNA sequences**		
Target	Sequence 5'-3'	
shLuc fw	TGTTCTCCGAACGTGTCACGTTTCAAGAGAACGTGACACGTTCGGAGAACTTTTTTC	
shLuc rv	CGAGAAAAAAGTTCTCCGAACGTGTCACGTTCTCTTGAAACGTGACACGTTCGGAGAACA	
shTIMP1 fw	TGGTTGCTATCACTGATAGCTTTCAAGAGAAGCTATCAGTGATAGCAACCTTTTTTC	
shTIMP1 rv	TCGAGAAAAAAGGTTGCTATCACTGATAGCTTCTCTTGAAAGCTATCAGTGATAGCAACCA	

shCD63 fw	TGGATTCTTGCTGCATCAACATATTCAAGAGATATGTTGATGCAGCAAGAATCCTTTTTTC	
shCD63 rv	TCGAGAAAAAAGGATTCTTGCTGCATCAACATATCTCTTGAATATGTTGATGCAGCAAGAATCCA	

Barragan, A., and L.D. Sibley. 2002. Transepithelial migration of Toxoplasma gondii is linked to parasite motility and virulence. *J Exp Med* 195:1625-1633.

Boyle, J.P., J.P. Saeij, and J.C. Boothroyd. 2007. Toxoplasma gondii: inconsistent dissemination patterns following oral infection in mice. *Exp Parasitol* 116:302-305.

Braun, L., M.P. Brenier-Pinchart, P.M. Hammoudi, D. Cannella, S. Kieffer-Jaquinod, J. Vollaire, V. Josserand, B. Touquet, Y. Couté, I. Tardieux, A. Bougdour, and M.A. Hakimi. 2019. The Toxoplasma effector TEEGR promotes parasite persistence by modulating NF-κB signalling via EZH2. *Nat Microbiol* 4:1208-1220.

Braun, L., M.P. Brenier-Pinchart, M. Yogavel, A. Curt-Varesano, R.L. Curt-Bertini, T. Hussain, S. Kieffer-Jaquinod, Y. Coute, H. Pelloux, I. Tardieux, A. Sharma, H. Belrhali, A. Bougdour, and M.A. Hakimi. 2013. A Toxoplasma dense granule protein, GRA24, modulates the early immune response to infection by promoting a direct and sustained host p38 MAPK activation. *J Exp Med* 210:2071-2086.

Gay, G., L. Braun, M.P. Brenier-Pinchart, J. Vollaire, V. Josserand, R.L. Bertini, A. Varesano, B. Touquet, P.J. De Bock, Y. Coute, I. Tardieux, A. Bougdour, and M.A. Hakimi. 2016. Toxoplasma gondii TgIST co-opts host chromatin repressors dampening STAT1-dependent gene regulation and IFN-gamma-mediated host defenses. *J Exp Med* 213:1779-1798.

Huynh, M.H., and V.B. Carruthers. 2009. Tagging of endogenous genes in a Toxoplasma gondii strain lacking Ku80. *Eukaryot Cell* 8:530-539.

Jensen, K.D., Y. Wang, E.D. Wojno, A.J. Shastri, K. Hu, L. Cornel, E. Boedec, Y.C. Ong, Y.H. Chien, C.A. Hunter, J.C. Boothroyd, and J.P. Saeij. 2011. Toxoplasma polymorphic effectors determine macrophage polarization and intestinal inflammation. *Cell Host Microbe* 9:472-483.

Kim, K., M.S. Eaton, W. Schubert, S. Wu, and J. Tang. 2001. Optimized expression of green fluorescent protein in Toxoplasma gondii using thermostable green fluorescent protein mutants. *Mol Biochem Parasitol* 113:309-313.

Kim, S.K., A. Karasov, and J.C. Boothroyd. 2007. Bradyzoite-specific surface antigen SRS9 plays a role in maintaining Toxoplasma gondii persistence in the brain and in host control of parasite replication in the intestine. *Infection and immunity* 75:1626-1634.

Mukhopadhyay, D., D. Arranz-Solís, and J.P.J. Saeij. 2020. Toxoplasma GRA15 and GRA24 are important activators of the host innate immune response in the absence of TLR11. *PLoS pathogens* 16:e1008586.

Sangare, L.O., E.B. Olafsson, Y. Wang, N. Yang, L. Julien, A. Camejo, P. Pesavento, S.M. Sidik, S. Lourido, A. Barragan, and J.P.J. Saeij. 2019. In Vivo CRISPR Screen Identifies TgWIP as a Toxoplasma Modulator of Dendritic Cell Migration. *Cell Host Microbe* 26:478-492 e478.

Schneider, C.A., W.S. Rasband, and K.W. Eliceiri. 2012. NIH Image to ImageJ: 25 years of image analysis. *Nature methods* 9:671-675.

Wang, Y., K.M. Cirelli, P.D.C. Barros, L.O. Sangare, V. Butty, M.A. Hassan, P. Pesavento, A. Mete, and J.P.J. Saeij. 2019. Three Toxoplasma gondii Dense Granule Proteins Are Required for Induction of Lewis Rat Macrophage Pyroptosis. *mBio* 10:

Figure S1

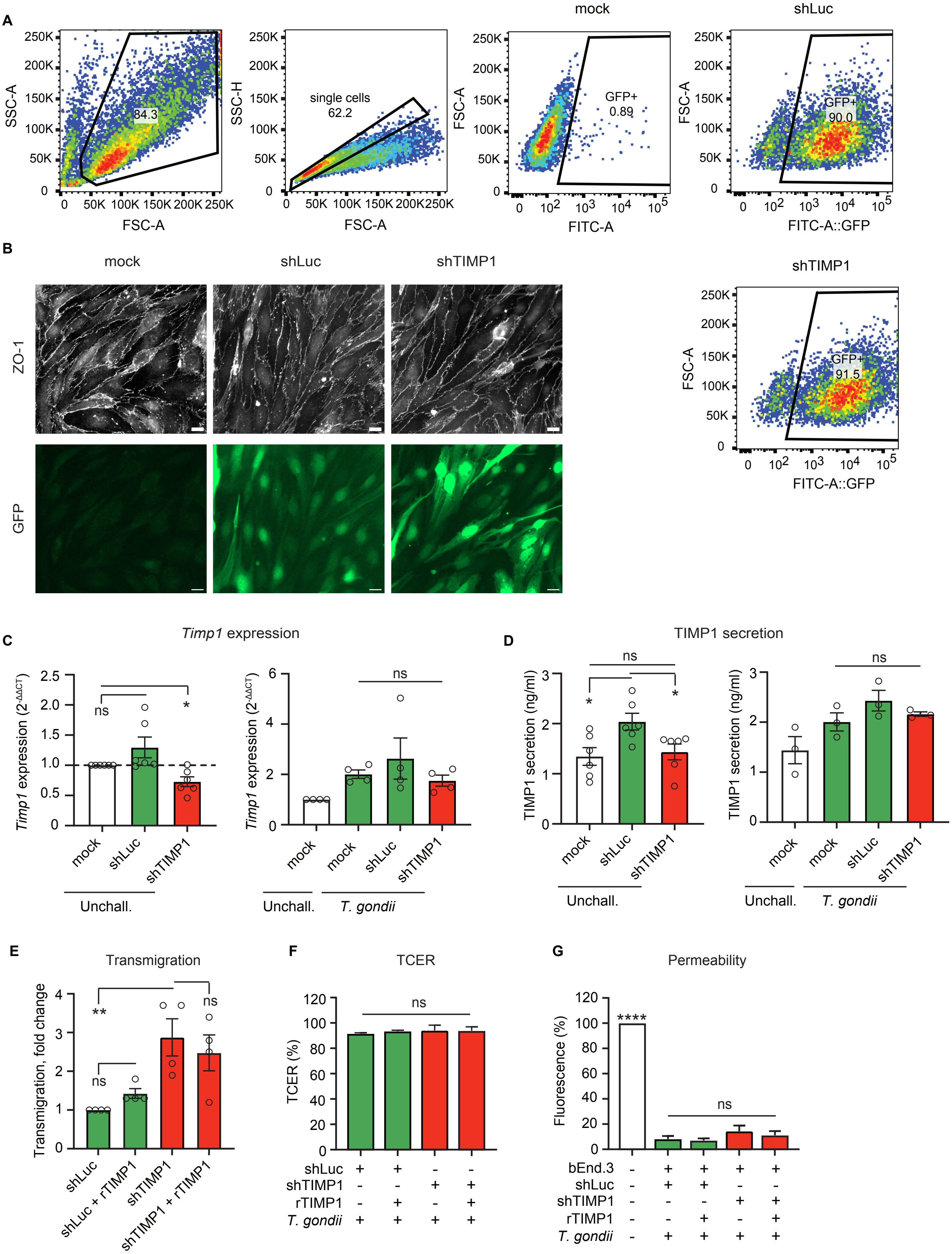
A
mock
shLuc
shTIMP1
SSC-A
SSC-H
FSC-A
FITC-A
FITC-A::GFP
84.3
single cells
62.2
GFP+
0.89
GFP+
90.0
GFP+
91.5
B
mock
shLuc
shTIMP1
ZO-1
GFP
C
Timp1 expression
Timp1 expression (2^-ΔΔCT)
ns
*
ns
mock
shLuc
shTIMP1
Unchall.
T. gondii
D
TIMP1 secretion
TIMP1 secretion (ng/ml)
ns
*
*
ns
mock
shLuc
shTIMP1
Unchall.
T. gondii
E
Transmigration
Transmigration, fold change
**
ns
ns
shLuc
shLuc + rTIMP1
shTIMP1
shTIMP1 + rTIMP1
F
TCER
TCER (%)
ns
shLuc
shTIMP1
rTIMP1
T. gondii
G
Permeability
Fluorescence (%)
****
ns
bEnd.3
shLuc
shTIMP1
rTIMP1
T. gondii

Figure S1. Impact of shRNA-mediated *Timp1* knockdown on TIMP1 expression, secretion and *T. gondii* transmigration

(**A**) Flow cytometry gating strategy for analysing mock-treated, shLuc- or shTIMP1-transduced bEnd.3 cell populations sorted after transduction. Representative plots show cell identification based on scatter-A/side scatter-A (from left, first panel), followed by single cell (second panel) analysis and percentage of GFP-expressing cells for the different conditions after cell sorting (mock, vector control shLuc and shTIMP1, respectively). Sorted cells were used in the assays. Representative data from 3 independent experiments.

(**B**) Representative micrographs of bEnd.3 cell monolayers mock-treated, transduced with GFP-expressing shLuc control plasmid or GFP-expressing shTIMP1 plasmid and stained for the TJ protein ZO-1 (grayscale, upper panel). Lower panel show GFP expression. Scale bars = 20 µm.

(**C**) Expression of *Timp1* and *Timp2* mRNA in mock-transduced, shTIMP1- or control shLuc-transduced bEnd.3 cells unchallenged or upon infection with *T. gondii*. Displayed as fold change ($2^{-\Delta\Delta Ct}$) in relation to the unchallenged mock-transduced condition.

(**D**) Abundance of TIMP1 polypeptide in supernatants of mock-treated, shTIMP1- or control shLuc-transduced bEnd.3 cells unchallenged or upon infection with *T. gondii*, determined by ELISA.

(**E**) Transmigration of *T. gondii* tachyzoites (PRU A7) across shLuc- or shTIMP1-transduced polarized bend.3 cell monolayers in presence of recombinant TIMP1 protein (rTIMP1, 100ng/ml), displayed as fold change in relation to transmigration of *T. gondii* tachyzoites across shLuc-transduced cell monolayers.

(**F**) Transcellular electrical resistance (TCER) mock-treated, shLuc- or shTIMP1-transduced bEnd.3 cell monolayers after *T. gondii* transmigration. Data are shown as % TCER ($\Omega \bullet cm^2$) relative to TCER values at the initiation of the assay (100%).

(**G**) Permeability to FITC-dextran (3 kDa) of mock-treated, shLuc- or shTIMP1-transduced bEnd.3 cell monolayers. Signal, measured as arbitrary fluorescence units (AU), from leaked FITC-dextran in lower transwell chamber was recorded at the end of the transmigration assay and related to signal of transwell insert in the absence of a polarized monolayer (100%).

Data are presented as mean (±SEM) from 3-6 independent experiments per condition. Statistical analyses were performed with one-way ANOVA, Sidaks post-hoc test, **P*<0.05, ***P*<0.01, *****P*<0.001, ns: non-significant.

Figure S2

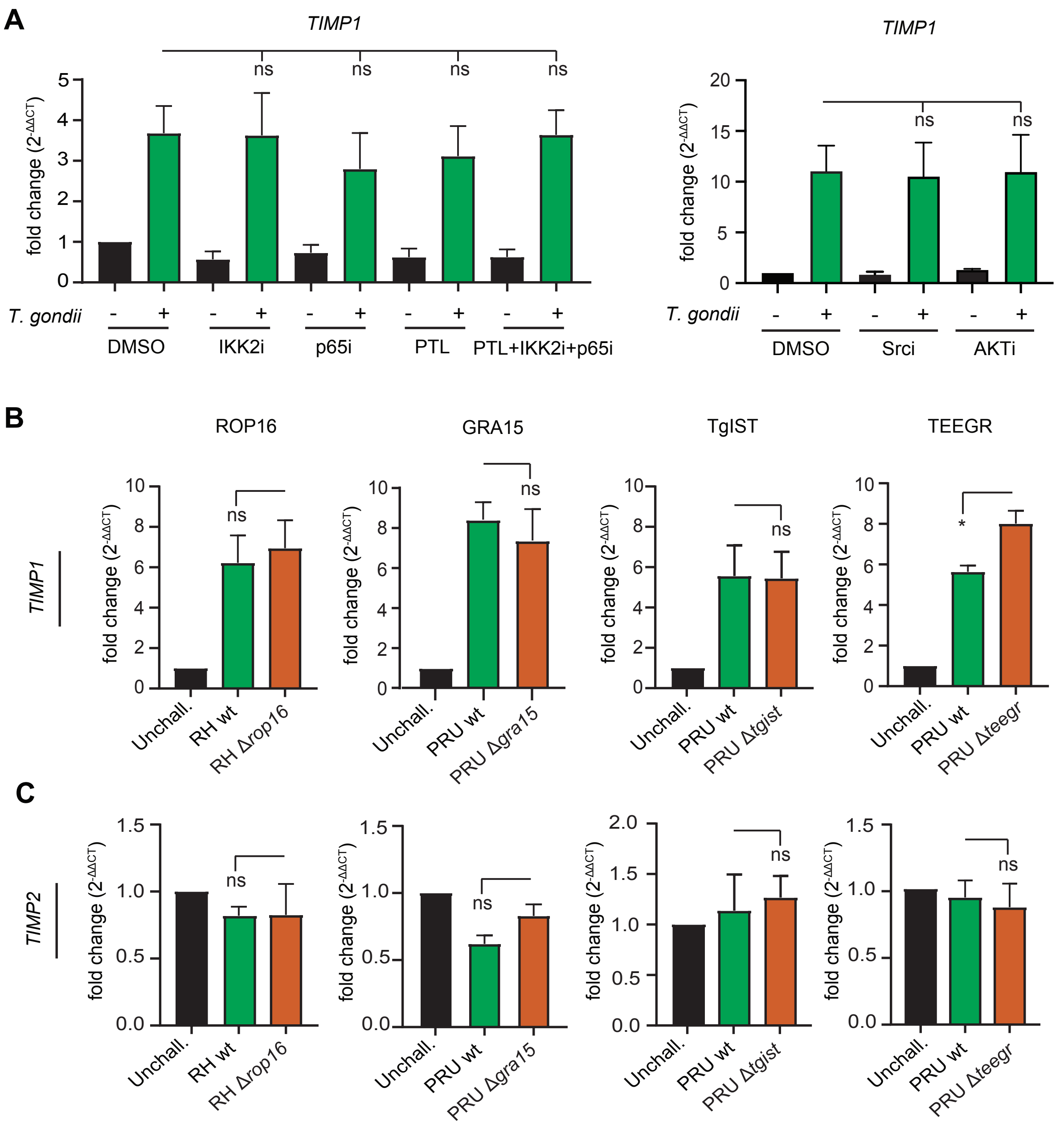

A
TIMP1
ns
ns
ns
ns
fold change (2-ΔΔCT)
T. gondii
- + - + - + - + - +
DMSO
IKK2i
p65i
PTL
PTL+IKK2i+p65i
TIMP1
ns
ns
- + - + - +
DMSO
Srci
AKTi
B
ROP16
GRA15
TgIST
TEEGR
TIMP1
ns
ns
ns
*
Unchall.
RH wt
RH Δrop16
PRU wt
PRU Δgra15
PRU Δtgist
PRU Δteegr
C
TIMP2
ns
ns
ns
ns

Figure S2. Effects of pharmacological inhibitors and *T. gondii* effector mutants (GRA15, ROP16, TgIST and TEEGR) on *TIMP1* expression

(**A**) qPCR analyses of *TIMP1* and *TIMP2* cDNA from Caco-2 cells challenged with *T. gondii* tachyzoites (RH LDM, MOI 2) for 6h in presence of NF-kB inhibitors: JSH23 (p65i, 20 µM), TPCA1 (IKK2i, 1 µM), parthenolide (PTL, 15 µM) or combination of all three, Src inhibitor 1 (Srci, 10 µM), Akt inhibitor MK2206 (Akti, 6 µM) (C) or DMSO as a vehicle control. Displayed as fold change ($2^{-\Delta\Delta Ct}$) in relation to unchallenged condition.

(**B, C**) qPCR analyses of *TIMP1* (**B**) and *TIMP2* (**C**) cDNA from Caco-2 cells challenged for 6h (MOI2) with *T. gondii* type I RH Ku80 (wild type) and ROP16-deficient mutant (Δ*rop16*), type II PRU A7 (wild type) and GRA15-deficient mutant (Δ*gra15*), PRU Ku80 (wild type), TgIST-deficient mutant (Δ*tgist*) and TEEGR-deficient mutant (Δ*teegr*).

Data are presented as mean (±SEM) from 3-4 independent experiments and displayed as fold change ($2^{-\Delta\Delta Ct}$) in relation to unchallenged condition. Statistical comparisons were performed with One-way ANOVA, Sidaks post-hoc test (**A**) or Student's *t*-test (**B**, **C**), **P* <0.05, ns: non-significant.
